# Activity engagement and cognitive function among chinese older adults: moderating roles of gender and age

**DOI:** 10.1186/s12877-023-03912-3

**Published:** 2023-04-06

**Authors:** Shan Mao, Lili Xie, Nan Lu

**Affiliations:** 1 Department of Social Work and Social Policy, School of Sociology and Population Studies, Renmin University of China, Beijing, China; 2 Center for Population and Development Studies, Renmin University of China, Room 604, Chongde Building, No. 59, Zhongguancun Street, Haidian District, Beijing, 100872 China; 3 School of Interdisciplinary Studies, Renmin University of China, Beijing, China

**Keywords:** Cognitive function, Activity engagement, Activity intensity, Active aging, Older adults

## Abstract

**Background:**

Many studies have found that engaging in activities, including physical exercise, social interaction, and cognitive training, is beneficial for preventing cognitive decline among older adults; however, the demographic differences in the association between activity engagement and cognitive functions remain understudied. This study investigates: (a) the influence of activity engagement on cognitive functions among Chinese older adults, and (b) the moderating roles of age and gender in these associations .

**Methods:**

The data were derived from the China Health and Retirement Longitudinal Study in 2018, which included 9803 participants aged 60 or older. A multiple regression model was used to test the study hypotheses.

**Results:**

Engaging in physical activity (*b* = 1.578, *p* < .001), social interaction (*b* = 1.199, *p* < .001), and cognitive activity (*b* = 1.468, *p* < .001) was positively associated with cognitive functions, whereas the effect of volunteer activities on cognitive functions was not significant (*b* = -.167, *p* = .390). Light- and moderate-intensity activities were beneficial for cognition (light: *b* = .847, *p* < .001; moderate: *b* = 1.189, *p* < .001), but vigorous-intensity activity was negatively related to cognition (*b* = -.767, *p* < .001). In addition, women and participants with advanced age appeared to benefit more from cognitive activities than their male and younger counterparts, respectively (gender: *b* = 1.217, *p* = .002; age: *b* = .086, *p* = .004). The adverse effects of vigorous-intensity activities (including agricultural work) on cognitive health were stronger for women and younger participants (gender: *b* = -1.472, *p* < .001; age: *b* = .115, *p* < .001). The protective effects of moderate-intensity activities on people’s cognition increased with increasing age (*b* = .055, *p* = .012).

**Conclusions:**

The findings indicate that participating in physical, social, and cognitive activities can help older adults to maintain cognitive health. They suggest that older adults should select activities while considering activity intensity and their individual characteristics.

**Supplementary Information:**

The online version contains supplementary material available at 10.1186/s12877-023-03912-3.

## Introduction

Cognitive health is an important component of successful aging [1]. Except normal cognitive aging, cognitive decline has other two deeper development stages: mild cognitive impairment and dementia. China has the fastest growing number of residents with cognitive impairment worldwide [2]. A recent study reported that approximately 39 million older adults experience mild cognitive impairment in China [3], and this number is estimated to reach 48.68 million by 2060 [2]. Cognitive impairment not only increases the risk of disability and mortality and makes older adults more dependent in daily life, but it also imposes huge care and economic burdens on families and society [4]. Cognitive decline may not always lead to dementia, and mild cognitive impairment is not completely irreversible [5]; however, mild cognitive impairment remains an important predictor of dementia [6], which has no cure. Therefore, identifying modifiable factors is crucial to preventing and delaying cognitive decline. It could also prolong older adults’ health span and promote their well-being later in life.

Previous studies have examined various determinants of cognitive functions, including demographic factors (e.g., age, gender), place of residence, socioeconomic status (e.g., education, income), physical health (e.g., activity of daily living, chronic diseases), and mental well-being (e.g., anxiety) [2, 3, 7, 8]. Other than these relatively stable characteristics, activity engagement, such as physical exercise, was also found to be beneficial for cognitive functions [9, 10]. According to the social model of health promotion, participating in physical, social, and cognitive activities can lead to better health outcomes [11, 12]. Prior evidence has indicated that these activities are associated with better cognitive performance [2, 13, 14] and may help to protect older adults’ cognition by maintaining brain function (e.g., increasing neural plasticity), improving physical and mental health (e.g., cardiovascular and cerebrovascular function, muscle strength, reduced depression), and expanding social networks [15–17]. Owing to the modifiable nature of lifestyle factors, studies examining the contribution of physical, social, and cognitive activities to cognitive health could provide valuable guidance regarding policies and interventions aimed at protecting older adults’ cognitive functions.

Although abundant studies have examined the association between activity participation and cognitive function from many perspectives, such as activity types, frequency, and intensity, their findings have been mixed [13, 16, 18, 19]. In addition, many studies have examined the role of a single type of activity on cognition functions without considering the effects of other kinds of activities simultaneously. The differential and independent effects of physical, social, and cognitive activities on cognitive functions remain unclear. Moreover, previous studies have indicated that the benefits of activity engagement on health outcomes (e.g., cognitive functions) vary across demographic characteristics [16]; although few studies have explored age and gender differences in the association between activity engagement and cognition, the findings have been inconsistent [20, 21]. Therefore, to better understand the relationship between activity engagement and cognitive functions among older adults, this study aimed to investigate the effects of three types of activities (i.e., physical, social, and cognitive) and their intensity on cognitive functions among Chinese older adults, along with the moderating roles of gender and age in these associations.

### Theoreticalframework and hypotheses engagement and cognitive reserve

#### Activity engagement and cognitive reserve

The concept of reserve (cognitive domains) was proposed to account for the discrepancy between brain damage and clinical cognitive performance [22–24]. Reserve comprises two important constructs: brain reserve and cognitive reserve. Brain reserve refers to the neurobiological capital of the brain (hardware), such as the number of neurons and synapses and cortical thickness [24, 25]. Individuals with greater initial brain mass can tolerate more neurological attrition before cognition is affected [26]. Cognitive reserve refers to the adaptability and plasticity of cognitive networks (software), and it can help the brain to operate effectively via neural reserve or compensation when cognitive impairment occurs [24]. Certain experiences and lifestyle factors, including education, job attainment, and leisure activities, were found as beneficial for brain maintenance and cognitive reserve development [23, 27]. Similar to the reserve concept, the Scaffolding Theory of Aging and Cognition argued that neural degradation, including neural challenges (e.g., cortical thickness) and functional deterioration (e.g., network connectivity), will stimulate “compensatory scaffolding”—a form of positive plasticity that accompanies aging—which could in turn reduce the adverse effects of neural and functional decline on cognitive performance [25]. Various lifestyle activities, such as exercise and cognitive training, play important roles in enhancing compensatory scaffolding. Accordingly, activity engagement may influence older adults’ cognitive health positively by protecting the brain and cognitive reserve or enhancing compensatory scaffolding.

#### Activity type and cognitive function

Physical activity can be defined as “any bodily movement produced by skeletal muscles that results in energy expenditure” (p. 126) [28]. Prior studies have shown that engaging in physical activities, including aerobic exercises (e.g., swimming, running), resistance training, and traditional Chinese exercises (e.g., qigong, tai ji quan), is associated with a lower risk of cognitive impairment and dementia among older adults [13, 29, 30]. Longitudinal studies have also reported that physical activities at midlife can reduce the long-term risk of dementia [31, 32]. Epidemiological research indicated that physical activity could protect cognitive functioning by reducing the risk of brain mass loss (gray and white matter) and chronic diseases [13, 15, 31].

Social activity is characterized by interactions with others [33]. Existing studies have suggested that older adults with higher frequency and diversity of social activity participation were more likely to have better cognitive performance [2, 16, 34, 35]. Both formal (i.e., participation in organized social activities) and informal (i.e., visits and interactions with friends) social participations were positively associated with cognitive functions among older adults, especially in rural areas [7].

A cognitive activity is an activity that requires mental response from participants [36]. Empirical evidence shows that cognitive activity can help older adults improve their cognitive reserve and cope with cognitive decline [14, 37, 38]. Although extensive literature has shown that physical, social, and cognitive activities are associated with better cognitive functions, other studies have reported non-significant results [19, 39]. The latter may be attributed to the different study populations and limited activity indicators adopted in some studies. In addition, frequent activity participation may also lead to risk of interpersonal conflicts, thereby increasing psychological stress, which has adverse effects on people’s health [16].

#### Activity intensity and cognitive function

Overall, different activities benefit cognitive functioning; however, the outcomes may vary because of different exertion levels. Intensity can be evaluated using both objective (e.g., using triaxial accelerometer) and subjective (self-reported) measures. It is generally classified into two or three levels based on certain criteria, including heart rate, breath, sweat, and duration [18, 40, 41]. Empirical evidence has shown that light, moderate, and vigorous intensity activities are positively associated with older adults’ cognitive health [17, 18, 40, 41]. Some studies show that moderate and vigorous intensity activities may have greater benefits for cognitive functioning than light-intensity activities [30, 40, 42]. Based on data from four provinces in China, a study found that excessive light-intensity exercise (10.5 to 21 h per week) was negatively associated with cognitive functioning [43], whereas another study reported the opposite results [18]. Besides having different measures and sample sizes, these mixed results also indicate potential demographic differences in the associations between activity types and intensities on cognitive functions.

#### Moderating roles of gender and age

Studies have indicated that the influence of activity participation on cognitive functions among older adults varies across genders and ages [16, 21, 42, 44, 45]. This may be partially attributed to the fact that people of different ages and genders have different preferences (hobbies) and motives for activity participation [46, 47]. For example, previous studies have suggested that women engage in more light-intensity activities, whereas men exert more energy in moderate-intensity activities, and the influence of physical exercise on cognition was stronger among older men than among women [48]. In addition, engaging in more social groups was associated with reduced cognitive decline among older women, whereas the effects was not significant among older men [35]. Women tended to have lower education levels than men did, which is an important protective factor for cognitive functions [2]. Cognitive and social activities may buffer the risk of cognitive decline among women [49]. Furthermore, some studies showed that the effects of physical exercise on cognitive functions (e.g., verbal fluency, memory recall) became more pronounced among older adults as they aged [45, 50].

Following theories and current evidence on activity engagement and cognitive functions, we proposed the following research hypotheses:

1. Engaging in physical, social, and cognitive activity is associated with better cognitive functions among Chinese older adults.
2. Activity intensities are associated with cognitive functions among Chinese older adults.
3. Gender moderates the association between physical, social, and cognitive activity and cognitive functions among Chinese older adults.
4. Age moderates the association between physical, social, and cognitive activity and cognitive functions among Chinese older adults.
5. Gender moderates the association between activity intensities and cognitive functions among Chinese older adults.
6. Age moderates the association between activity intensities and cognitive functions among Chinese older adults.

## Method

### Sampling

The data were derived from the fourth wave of China Health and Retirement Longitudinal Study, conducted by National School of Development (China Centre for Economic Research) of Peking University in 2018. Using a multistage stratified cluster random sampling method, 19,816 individuals aged 45 or older (main respondents and spouses) from 10,524 households in 450 village-level units, 150 county-level units, and 28 provinces, completed surveys, with a response rate of 83.84%. For more details about survey design and data collection, please refer to the user’s guide [51].

This study focused on older adults. Thus, 10,761 respondents aged 60 or older were selected as the study sample (8,639 were aged 59 or younger, and 317 had missing data for age). After excluding participants with missing data on cognitive functions (*n* = 958), the final sample size was 9,803. The data were obtained on request through the original study’s official website (http://charls.pku.edu.cn/). This secondary data analysis did not require additional ethics approval.

### Measurements

#### Cognitive function

Cognitive function was measured by orientation, episodic memory, calculation, language, naming, and visuospatial ability. The first five cognitive domains were assessed using items derived from a modified Chinese version of the Telephone Interview of Cognitive Status [52, 53]. Orientation was assessed by asking respondents to state the year, month, date, day of the week, and season (1 point for each correct answer). Episodic memory included immediate and delayed recall. To test immediate recall, the interviewer read 10 Chinese nouns and the respondents recalled them immediately. A few minutes later, respondents were asked to recall these words again to assess delayed recall. The calculation involved five-time serial subtraction of 7 from 100, with scores ranging from 0 to 5. To assess the language, respondents were required to repeat a phrase after the interviewer, which is similar to “no ifs, ands, or buts”. Naming was evaluated by asking respondents the following three questions: What do people usually use to cut paper? What do you call the kind of prickly plant that grows in the desert? Who is the president of China right now? Visuospatial ability was measured by figure drawing. Respondents were required to draw two overlapping pentagons similar to a picture shown by the interviewer (1 = *correct*, 0 = *error*). A sum score reflected the respondents’ cognitive status, ranging from 0 to 35. Higher scores indicated better cognitive health.

#### Activity engagement

Activity engagement was measured by asking respondents whether they engaged in any of 11 activities in the past month. Answers to each question were coded as a binary variable (1 = *yes*, 0 = *no*). Social activities included social interaction (interpersonal activities) and volunteering. Social interaction was assessed using four items: visiting friends, participation in community activity, using the internet, and other social interactions. Volunteering was measured using three items: helping people (e.g., family, friends, and neighbors) who did not live with the respondent, taking care of sick or disabled adults who did not live with the respondent, as well as formal charity or voluntary work. Cognitive activity was measured using three items: board games (e.g., mah-jongg, cards, and chess), educational or training courses, and stock investment. Total scores were calculated separately to present participation levels in social interaction, volunteering, and cognitive activity. Physical activity was measured using a single item: participating in physical-exercise groups such as dancing, qigong, fitness, and so on (1 = *yes*, 0 = *no*).

#### Activity intensity

Activity intensity was assessed using three subjective items. Specifically, vigorous-intensity activity was measured by asking respondents whether they participate in activities that can cause shortness of breath (e.g., carrying heavy stuff, hoeing, digging, and bicycling at a fast speed) for at least 10 min every week. Moderate-intensity activity was measured by asking respondents whether they participate in activities that could make them breathe faster than usual (e.g., carrying light stuff, bicycling at a normal speed, speed walking) for at least 10 min every week. Light-intensity activity was assessed by asking respondents whether they participate in activities in which they can maintain normal breathing (e.g., walking at a normal speed) for at least 10 min every week. All answers were recoded as binary variables (1 = *yes*, 0 = *no*).

#### Control variables

Control variables in this study included age (in years), gender (0 = *male*, 1 = *female*), education (0 = *primary school or below*, 1 = *secondary school or above*), marital status (1 = *married*, 0 = *other*), self-reported annual individual income (sum of salary and pensions, log value), residence (0 = *urban*, 1 = *rural*), self-reported health, and activities of daily living (ADL). Self-reported health was measured by asking respondents to provide an overall evaluation of their health status, with answers coded as 1 (*very poor*), 3 (*fair*), and 5 (*very good*). ADL was measured by asking respondents whether they experience difficulty in eating, dressing, bathing, toileting, defecating, and getting in and out of bed [54]. Responses were recoded as 1 (*having difficulties*) and 0 (*having no difficulties*). Age and gender were also treated as moderators in the last moderating model.

### Data analysis

Stata 16.0 was used to build multiple regression models to examine research hypotheses. First, covariates including age, gender, education, individual income, marital status, residence, self-reported health, and activities of daily living, were regressed on cognitive function in Model 1. Second, activity type and intensity variables were regressed on cognitive function separately to test Hypotheses 1 and 2 (Models 2 and 3). Third, corresponding two-way interaction terms were entered into Models 2 and 3 to test Hypotheses 3 to 6 (Models 4 and 5). Finally, we conducted a sensitive analysis to test the robustness of results: All independent variables (activity types and intensities) were added to the same model to examine their main effects on cognition. Thereafter, all interaction items were added to this model to test the moderating effects of age and gender. A robust regression method was adopted to estimate regression coefficients. Estimates of the variance inflation factor were used to test multicollinearity in the regression models [55], and the results indicated no multicollinearity.

## Results

### Descriptive characteristics

Table 1 presents the characteristics of the study sample. The average age of the 9,803 respondents was 68.68 years, and 50.58% were female. More than 70% of the respondents were living in rural areas, approximately 80% were married; and 29.09% did not receive any formal education (i.e., illiteracy). Respondents’ average individual annual income was approximately 10,068 RMB (about $1,522 USD in 2018). Regarding physical health, 48.12% of respondents reported that they had a fair health status and 69.36% had no physical limitations in daily life. The average score of cognitive function was 13.13. During the past month, 35.36% of participants interacted with friends, 12.38% participated in volunteer activities, 17.55% engaged in cognitive activities, and only 5.68% participated in sports groups; additionally, 44.68% had participated in moderate-intensity activities in the last week.

**Table 1:** Sample characteristics (*N* = 9803)

Variables	Percentage (%)/Means (SD)	Variables	Percentage (%)/Means (SD)
**Age (60–108)**	68.68 (6.57)	**Cognitive function (0–34)**	13.13 (7.42)
60–69 years	6114 (62.37)	**Physical activity**	
70 years or above	3689 (37.63)	*Yes*	577 (5.68)
**Gender** (%)		*No*	9244 (94.32)
*Female*	4958 (50.58)	**Social interaction**	
*Male*	4845 (49.42)	*Yes*	3466 (35.36)
**Residence** (%)		*No*	6335 (64.64)
*Rural*	7192 (26.63)	**Volunteering**	
*Urban*	2611 (73.37)	*Yes*	1213 (12.38)
**Marital status** (%)		*No*	8588 (87.62)
*Married*	7826 (79.83)	**Cognitive activity**	
*Other status*	1977 (20.17)	*Yes*	1720 (17.55)
**Education level** (%)		*No*	8081 (82.45)
*Primary school and below*	7280 (74.26)	**Light-intensity activity**	
*Secondary school and above*	2523 (25.74)	*Yes*	7993 (81.54)
**Individual income (RMB)**	10,068.46 (19,872.38)	*No*	1809 (18.46)
**Self-reported health** (1–5)	2.94 (1.01)	**Moderate-intensity activity**	
*Poor or very poor*	3002 (30.65)	*Yes*	4380 (44.68)
*Fair*	4713 (48.12)	*No*	5422 (55.32)
*Good or very good*	2080 (21.23)	**Vigorous-intensity activity**	
**Activities of daily living (ADL)**	0.34 (0.62)	*Yes*	2644 (26.97)
*Having difficulty*	2334 (30.64)	*No*	7158 (73.03)
*Having no difficulty*	5284 (69.36)		

### Results of multiple regression analysis

Table 2 shows the main effects of activity engagement on cognitive functions and the gender and age differences in these associations. Model 1 shows that education, income, and being married were factors protecting cognitive functions; however, age was negatively associated with cognitive functions. Older men appeared to have better cognitive condition than older women. Model 2 results suggest that social interaction, physical activity, and cognitive activity were positively associated with cognitive functions, whereas the effect of volunteering on cognitive functions was not significant. Model 3 shows that both light-and moderate-intensity activities were beneficial for older adults’ cognitive health. However, vigorous-intensity activity was found as negatively related to cognitive functions.

**Table 2:** Results of multiple regression analysis

Independent variables	Model 1			Model 2			Model 3			Model 4			Model 5		
	B (S.E.)	β	P-value	B (S.E.)	β	P-value	B (S.E.)	β	P-value	B (S.E.)	β	P-value	B (S.E.)	β	P-value
Age (60 ~ 118)	-.168 (.011)	-.153	.000	-.159 (.011)	-.144	.000	-.159 (.011)	-.144	.000	-.154 (.011)	-.140	.000	-.141 (.011)	-.128	.000
Gender (1 = female)	-1.467 (.152)	-.098	.000	-1.584 (.151)	-.106	.000	-1.634 (.152)	-.109	.000	-1.542 (.151)	-.103	.000	-1.622 (.152)	-.109	.000
Education	5.562 (.183)	.311	.000	5.211 (.184)	.291	.000	5.481 (.182)	.307	.000	5.246 (.185)	.293	.000	5.523 (.182)	.309	.000
Individual income	.261 (.022)	.122	.000	.236 (.022)	.111	.000	.248 (.022)	.116	.000	.232 (.022)	.109	.000	.244 (.022)	.114	.000
Marital status	1.480 (.176)	.083	.000	1.538 (.174)	.086	.000	1.486 (.174)	.083	.000	1.533 (.174)	.086	.000	1.459 (.175)	.082	.000
Residence (1 = rural)	-2.940 (.183)	-.173	.000	-2.608 (.183)	-.153	.000	-2.780 (.186)	-.164	.000	-2.594 (.183)	-.153	.000	-2.763 (.186)	-.163	.000
Self-reported health	.079 (.076)	.010	.302	.017 (.076)	.002	.823	.042 (.076)	.006	.578	.013 (.076)	.002	.859	.042 (.076)	.005	.580
Activities of daily living (ADL)	-.925 (.163)	-.057	.000	-.860 (.161)	-.053	.000	-.819 (.163)	-.051	.000	-.864 (.161)	-.054	.000	-.798 (.162)	-.050	.000
Social interaction				1.199 (.128)	.093	.000				1.014 (.185)	.079	.000			
Volunteering				-.167 (.195)	-.008	.390				-.400 (.307)	-.020	.192			
Cognitive activity				1.468 (.196)	.074	.000				.963 (.267)	.049	.000			
Physical activity				1.578 (.334)	.047	.000				764 (.566)	.023	.177			
Vigorous-intensity activity							-.767 (.176)	-.045	.000				.209 (.247)	.012	.397
Moderate-intensity activity							1.189 (.151)	.079	.000				1.057 (.221)	.071	.000
Light-intensity activity							.847 (.181)	.046	.000				.374 (.285)	.020	.189
Gender X social interaction										.332 (.247)	.019	.179			
Gender X volunteering										.467 (.387)	.018	.227			
Gender X cognitive activity										1.217 (.386)	.042	.002			
Gender X physical activity										.954 (.703)	.024	.175			
Age X social interaction										-.018 (.018)	-.009	.300			
Age X volunteering										.025 (.035)	.008	.486			
Age X cognitive activity										.086 (.030)	.027	.004			
Age X physical activity										-.003 (.052)	-.001	.961			
Gender X vigorous-intensity activity													-1.472 (.336)	-.062	.000
Gender X moderate-intensity activity													.271 (.298)	.014	.363
Gender X light-intensity activity													.695 (.363)	.028	.055
Age X vigorous-intensity activity													.115 (.028)	.040	.000
Age X moderate-intensity activity													.055 (.022)	.024	.012
Age X light-intensity activity													.043 (.023)	.017	.059
R^2^	.3018			.3204			.3109			.3228			.3160		
Adjusted R^2^	.3010			.3193			.3099			.3210			.3144		
F value	514.44***			376.51			394.37***			233.63***			267.66***		
Mean VIF	1.15			1.14			1.15			1.81			1.61		

*B* unstandardized coefficients, *S.E.* (robust) standardized error, *β* standardized coefficients, *VIF* Variance Inflation Factor; ***p < .001. For model 1, only control variables were regressed on cognitive function; For model 2, control variables and activity type variables were regressed on cognitive function; For model 3, control variables and activity intensity variables were regressed on cognitive function; For model 4, control variables, activity type variables, interaction terms of age and activity types, and interaction terms of gender and activity types were regressed on cognitive function; For model 5, control variables, activity intensity variables, interaction terms of age and activity intensities, and interaction terms of gender and activity intensities were regressed on cognitive function

Regarding the moderating effects, Model 4 showed that gender and age played moderating roles in the association between cognitive activity and functions. Women and older participants seemed to benefit more from cognitive activities than their male and younger counterparts, respectively. Model 5 showed that the negative effects of vigorous-intensity activity on cognitive function were stronger for women and younger participants than for men and older participants, respectively. Moderate-intensity activity had a greater protective effect on cognitive function for older respondents than for their younger counterparts. However, the moderating effects of age and gender in the relationships between physical activity, social activity (i.e., social interaction, volunteering), and light-intensity activity and cognitive function were not significant. The sensitivity analysis indicated similar results. For more details, please refer to the table in Additional file 1 : Appendix. Figures plotting the significant interactions were also presented in Additional file 1 : Appendix.

## Discussion

To the best of our knowledge, this is among the first studies to explore the moderating mechanisms of three types of activities, activity intensity, age, gender, and cognitive functions among Chinese older adults. The study findings add new empirical evidence to support the reserve theory of cognition and provide valuable guidance for activity interventions to improve cognition among different groups of older adults.

The study results suggest that older adults who participate in physical activities (exercise), cognitive activity, and social interaction are more likely to have better cognitive performance, as supported by previous studies [14, 29, 49]. Different types of activities could influence individuals’ cognitive function through different pathways. Physical exercise may protect cognition by improving physical health and maintaining brain reserve (hardware; [13, 31]. Cognitive activity may increase cognitive reserve such as neural plasticity (software) via complex thinking and mental training [31, 37]. Social interaction can help maintain cognitive functions by improving mental health and encouraging healthy behaviors [7, 16]. In addition, the study results indicate that participating in volunteer and charity activities did not impact older adults’ cognitive health, which is contrary to many existing studies [16, 34]. This non-significant result may be explained by the small proportion of participants who engaged in volunteer activity. Conversely, volunteer work such as taking care of people with disabilities may impose physical and mental burdens on older adults, which could counteract its positive effect on health. A study found that both scarce and excessive participation in volunteer activities were negatively associated with older adults’ health [56]. Another study reported that volunteering was positively associated with psychosocial outcomes such as loneliness and depressive symptoms, but not associated with other health domains including cognitive impairment [57].

Regarding activity intensity, our research demonstrates that light and moderate intensity activities could help older adults maintain cognitive function, which is in line with previous findings [17, 40]. However, contrary to the conclusion of many prior studies, the current study showed that vigorous intensity activities were negatively associated with cognitive health [30, 42]. Further, previous studies mainly focused on vigorous physical exercise; however, the vigorous-intensity activities in our study also included heavy physical work (e.g., carrying heavy stuff, digging, and hoeing). Some research found that vigorous exercise during leisure time predicted good physical health, but strenuous physical work was negatively associated with physical function [58, 59]. In this study, more than 70% of the respondents were rural residents, who generally spend much time on agricultural work even in their old age. A prior study noted that engaging in agricultural work may increase the risk of cardiovascular disease, which is closely associated with cognitive decline [60]. Therefore, vigorous physical work may harm older adults’ cognitive functions by affecting their physical health. To verify this explanation further, we also examined the effects of vigorous-intensity activity on cognitive functions among rural and urban groups separately. The results show that vigorous-intensity activity had an adverse effect on cognition among rural residents, whereas urban residents participated less in vigorous-intensity activities, and the influence on cognition was not significant.

In line with previous studies, advanced age and female gender were risk factors of cognitive decline [3]. The faster cognitive decline among women may be partially explained by longer life expectancy, lower educational level, and biological difference (e.g., sex hormones difference, brain structure, genetics, gray volume loss) with men [3, 8]. Additionally, the current study found age and gender differences in the associations between activity engagement and cognitive functions among older adults. Regarding gender, cognitive activities had a greater effect on cognitive health for older women. Compared with men, women had lower educational attainment. Cognitive activities could work as alternative educational resources to increase women’s cognitive reserve and further prevent cognitive decline [35, 49]. Moreover, our findings show that the adverse impact of vigorous-intensity activities on cognition was also stronger for women than for men. A possible explanation is that women’s physical capacities (e.g., physical strength and muscles) are lower than those of men; therefore, the same physical load of vigorous-intensity activities may cause more damage for older women. Contrary to studies showing that the favorable effects of physical and social activities differed by gender [21, 44], our study suggests that the influence of physical activity and social interaction were equal among men and women in the Chinese context, echoing another Chinese study [34].

Regarding age, the findings indicate that older adults with advanced age could benefit more from cognitive activities and moderate-intensity activities than their younger counterparts. Studies have determined that continuous participation in cognitive activities can protect cognitive functions [35], and the cumulative protective effect of cognitive activities may be greater for older adults with advanced age. In addition, older adults’ health conditions tend to deteriorate as they age. Therefore, the benefits of cognitive and moderate-intensity activities for cognition could be more prominent with age [45, 50]. Moreover, the negative effect of vigorous activities among older adults attenuated with increasing age. There have two possible explanations: First, physical capacities decline with increasing age; older adults may be less likely to engage in vigorous physical exercise and work, especially rural residents. Second, those who in the advanced age still participating in vigorous-intensity activities might generally have better cognitive function even compared with some younger elderly.

This study highlights the importance of physical, social, and cognitive activities for older adults’ cognitive health as well as the roles for adults of different ages and genders. These findings provide valuable suggestions for social work practice to promote cognitive functions among Chinese older adults. Communities should provide more high-quality recreational facilities for older adults (e.g., senior centers and fitness equipment), launch hobby groups, and organize various activities, which can encourage older adults to participate in physical exercise and cognitive leisure activities regularly, help them build greater social networks, and provide more opportunities for social interactions. Social workers can cooperate with physicians or other professionals to design and implement intervention programs such as mind–body exercises to help older adults maintain cognitive functions. Older women and rural residents had a higher risk of cognitive decline; therefore, policy initiatives and intervention programs should also focus more on older women in rural areas. Given the different cognitive status and preferences of older adults, needs assessment including health evaluation should be conducted, which could help older adults choose appropriate activities, promote positive participation experiences, and increase the possibility of continuous participation in cognitively beneficial activities. Regarding activity intensity, although many studies reported that vigorous-intensity activity could improve cognitive health, other research, including the present study, drew different conclusions [58, 59]. The appropriate intensity and types of physical activities that are beneficial for cognition remain unclear. Owing to the comorbidity and fragility of older adults in later life, we recommend that older adults participate in light-and moderate-intensity activities (e.g., tai chi, walking) and reduce high-load physical work.

## Limitations

This study had some limitations. First, cross-sectional data cannot be used to infer the causal relationships between activity engagement and cognitive functions. Second, although we used a global measure of activity type, we included a limited number of activities due to the data limitation, and the dichotomous scale items provided limited information on activity engagement. In addition, physical activity participation may have been underestimated, because the measures only evaluated group-based physical exercise, and individual physical activity were not included. For activity intensities, although CHARLS used a revised version of International Physical Activity Questionnaire (IPAQ) to assess activity intensity [61, 62], we could not calculate the duration of different activity intensity levels because of the discrete time duration variables and the large number of missing data. And this study did not assess the intensities of different activity types separately (e.g., domestic activities, social activities, physical work, and physical exercise). If the above information had been considered, we might have gained a better understanding of the association between activity participation and cognitive functions, and the guidance provided by the study findings might have been more useful for policies and interventions. Third, both activity type and intensity were self-reported and might be affected by recall bias. Fourth, the activity classifications were not perfectly mutually exclusive. Certain activities have more than one nature. For example, participating in exercise groups has both physical and social elements; playing mah-jongg or card may be both intellectual and social. Fifth, compared with the computerized cognitive assessment batteries or clinical dementia screening tools, the TICS scale cannot evaluate individuals’ cognitive status in detail. Sixth, this study only examined the relationship between activity participation and total cognitive functions, without exploring the influence of activities on specific dimensions of cognition. Finally, we only examined the direct effects of activity engagement on cognition. Future studies may adopt a longitudinal design and more reliable and detailed measurements of activity participation to examine the relationship between different activities and subdomains of cognitive functions among specific groups. Given the nature of cognitive health, an interdisciplinary approach is recommended to explore the potential mechanisms of different activities and cognitive functions.

## Conclusions

Underpinned by nationwide representative data, this study examined the independent effects of physical activities, social interaction, volunteering, cognitive activities, and activity intensity on cognitive functions, along with the moderating roles of age and gender. The findings indicate that (a) physical activities, social interaction, cognitive activities, and light-to moderate intensity activities could help maintain cognitive functioning; (b) volunteer activities were not associated with cognitive functions; (c) vigorous-intensity activities had a negative influence on cognition, especially for women and younger participants; and (d) cognitive activities had greater cognitively benefits for women and older participants.

## Supplementary Information

**Additional file 1.** The sensitive analysis results of multiple regressionmodel.

## Data Availability

The datasets analyzed during the current study are available in the CHARLS repository: http://charls.pku.edu.cn/.

## Abbreviations

CHARLS: The China Health and Retirement Longitudinal Study
SD: Standard deviation
S.E.: Standardized error
β: Beta
ADL: Activities of Daily Living
