# Supplementary material for: Activity engagement and cognitive function among chinese older adults: moderating roles of gender and age
Source: BMC Geriatr. 2023 Apr 6;23:223. doi: 10.1186/s12877-023-03912-3 (PMC10080791; doi:10.1186/s12877-023-03912-3)
Supplement: Supplementary file 1 — Additional file 1. The sensitive analysis results of multiple regressionmodel. [file 12877_2023_3912_MOESM1_ESM.docx]

Appendix

Table S1 Sensitive analysis results of the multiple regression model

| Independent variables | Model 1 | | | Model 2 | | | Model 3 | | |
| --- | --- | --- | --- | --- | --- | --- | --- | --- | --- |
|  | B (S.E.) | β | P-value | B (S.E.) | β | P-value | B (S.E.) | β | P-value |
| Age (60~118) | -.168 (.011) | -.153 | .000 | -.153 (.011) | -.139 | .000 | -.134 (.011) | -.121 | .000 |
| Gender (1 = female) | -1.467 (.152) | -.098 | .000 | -1.711 (.151) | -.115 | .000 | -1.662 (.151) | -.111 | .000 |
| Education | 5.562 (.183) | .311 | .000 | 5.166 (.184) | .289 | .000 | 5.234 (.184) | .293 | .000 |
| Individual income | .261 (.022) | .122 | .000 | .227 (.022) | .106 | .000 | .223 (.022) | .104 | .000 |
| Marital status | 1.480 (.176) | .083 | .000 | 1.544 (.173) | .087 | .000 | 1.511 (.173) | .085 | .000 |
| Residence (1 = rural) | -2.940 (.183) | -.173 | .000 | -2.492 (.186) | -.147 | .000 | -2.468 (.186) | -.145 | .000 |
| Self-reported health | .079 (.076) | .010 | .302 | .007 (.075) | -.001 | .929 | -.010 (.075) | -.001 | .894 |
| Activities of daily living (ADL) | -.925 (.163) | -.057 | .000 | -.781 (.161) | -.049 | .000 | -.767 (.160) | -.048 | .000 |
| Vigorous-intensity activity |  |  |  | -.701 (.175) | -.041 | .000 | .243 (.247) | .014 | .325 |
| Moderate-intensity activity |  |  |  | .985 (.151) | .066 | .000 | 1.012 (.224) | .068 | .000 |
| Light-intensity activity |  |  |  | .690 (.179) | .037 | .000 | .311 (.281) | .017 | .269 |
| Social interaction |  |  |  | 1.114 (.128) | .087 | .000 | .967 (.184) | .075 | .000 |
| Volunteering |  |  |  | -.221 (.197) | -.011 | .261 | -.593 (.302) | -.030 | .050 |
| Cognitive activity |  |  |  | 1.417 (.196) | .072 | .000 | .934 (.266) | .047 | .000 |
| Physical activity |  |  |  | 1.346 (.335) | .040 | .000 | .453 (.564) | .013 | .423 |
| Gender*vigorous-intensity activity |  |  |  |  |  |  | -1.440 (.334) | -.060 | .000 |
| Gender*moderate-intensity activity |  |  |  |  |  |  | .021 (.300) | .001 | .944 |
| Gender*light-intensity activity |  |  |  |  |  |  | .550 (.361) | .023 | .127 |
| Gender*social interaction |  |  |  |  |  |  | .225 (.247) | .013 | .363 |
| Gender*volunteering |  |  |  |  |  |  | .676 (.388) | .026 | .082 |
| Gender*cognitive activity |  |  |  |  |  |  | 1.120 (.386) | .039 | .004 |
| Gender*physical activity |  |  |  |  |  |  | .938 (.705) | .024 | .183 |
| Age*vigorous-intensity activity |  |  |  |  |  |  | .107 (.028) | .037 | .000 |
| Age*moderate-intensity activity |  |  |  |  |  |  | .061 (.022) | .026 | .006 |
| Age*light-intensity activity |  |  |  |  |  |  | .038 (.023) | .015 | .097 |
| Age*social interaction |  |  |  |  |  |  | -.032 (.018) | -.016 | .078 |
| Age*volunteering |  |  |  |  |  |  | -.006 (.034) | -.002 | .872 |
| Age*cognitive activity |  |  |  |  |  |  | .080 (.030) | .025 | .008 |
| Age*physical activity |  |  |  |  |  |  | -.034 (.051) | -.006 | .510 |
| R^2^ | .3018 |  |  | .3266 |  |  | .3336 |  |  |
| Adjusted R^2^ | .3010 |  |  | .3253 |  |  | .3311 |  |  |
| F value | 514.44*** |  |  | 313.09*** |  |  | 174.64*** |  |  |
| Mean VIF | 1.15 |  |  | 1.15 |  |  | 1.90 |  |  |

Notes: B, unstandardized coefficients; S.E., (robust) standardized error; β, standardized coefficients; VIF, Variance Inflation Factor; ****p* < .001
